# Lifetime risk and health-care burden of diabetic ketoacidosis: A population-based study

**DOI:** 10.3389/fendo.2022.940990

**Published:** 2022-08-24

**Authors:** Fahim Ebrahimi, Alexander Kutz, Emanuel Remigius Christ, Gabor Szinnai

**Affiliations:** ^1^ University Center for Gastrointestinal and Liver Diseases, St. Clara Hospital and University Hospital, Basel, Switzerland; ^2^ Division of Endocrinology, Diabetes, and Metabolism, University Hospital Basel, Basel, Switzerland; ^3^ Department of Clinical Research, University Hospital Basel, University Basel, Basel, Switzerland; ^4^ Division of Endocrinology, Diabetes, and Metabolism, University Department of Medicine, Kantonsspital Aarau, Aarau, Switzerland; ^5^ Division of General Internal and Emergency Medicine, University Department of Medicine, Kantonsspital Aarau, Aarau, Switzerland; pt?> ^6^ Pediatric Endocrinology and Diabetology, University Children’s Hospital Basel, University of Basel, Basel, Switzerland

**Keywords:** hyperglycemic crisis, ketoacidosis (DKA), type 1 diabetes mellitus (T1D), type 2 diabetes mellitus, coma (diabetic)

## Abstract

**Objective:**

Diabetic ketoacidosis (DKA) is a life-threatening complication of both type 1 and type 2 diabetes. We aimed to assess population-based rates, trends and outcomes of patients with DKA.

**Design and methods:**

This is a nationwide cohort study using hospital discharge claims data from 2010 to 2018 in Switzerland. Incidence rates and in-hospital outcomes of DKA were analyzed throughout lifetime for children (0-9 years), adolescents (10-19 years), and adults (20-29, 30-59, and 60-90 years). Analyses were stratified for type of diabetes mellitus and sex.

**Results:**

In total, 5,544 hospitalizations with DKA were identified, of whom 3,847 were seen in patients with type 1 diabetes and 1,697 in type 2 diabetes. Incidence rates of DKA among patients with type 1 diabetes were highest during adolescence with 17.67 (girls) and 13.87 (boys) events per 100,000 person-years (incidence rate difference [IRD]: -3.80 [95% CI, -5.59 to -2.02]) and decreased with age in both sexes thereafter. Incidence rates of DKA in patients with type 2 diabetes were low up to an age of 40 years and rose to 5.26 (females) and 6.82 (males) per 100,000 person-years in adults aged 60-90 years. Diabetic ketoacidosis was associated with relevant health-care burden independent of age, sex, or type of diabetes. The population-based incidence rate of DKA increased over time from 7.22 per 100,000 person-years in 2010 to 9.49 per 100,000 person-years in 2018.

**Conclusions:**

In type 1 diabetes highest incidence rates of DKA hospitalizations were observed among adolescent females. In comparison, in patients with type 2 diabetes the risk for DKA steadily increased with age with higher rates in adult males. Over the 9 year study period, incidence rates of DKA were increasing irrespective of type of diabetes. DKA was associated with a high burden of disease reflected by high rates of intensive care unit admission, prolonged hospital stay and high mortality rates, especially in elderly.

## Introduction

Diabetic ketoacidosis (DKA) and hyperglycemic hyperosmolar state are life-threatening complications occurring at states of metabolic decompensation or at primary diagnosis of diabetes mellitus (1–3). While patients with type 1 diabetes are known to be susceptible to DKA particularly under stressful conditions such as infection, trauma, or surgery, ketoacidosis may also occur in patients with poorly controlled or newly diagnosed type 2 diabetes (4, 5). Especially in recent years, the broader use of sodium–glucose cotransporter 2 (SGLT2) inhibitors has raised awareness of DKA in type 2 diabetes, since this modern antidiabetic drug class precipitates euglycemic DKA (6).

There are disparities in occurrence of DKA, in that patients from disadvantaged socioeconomic backgrounds or with poor mental health have a higher incidence rates of DKA (7, 8). Although management of diabetes and its associated complications has significantly improved over the past decades (9, 10), DKA remains a significant health burden with high morbidity (11), mortality (12–14), and relevant utilization of health-care resources (15–17).

While recent data indicate that the population incidence for DKA has been increasing dramatically over the past years (18), data on risks throughout lifetime and granular characteristics of individuals at highest risk of DKA are scarce. Most published data about the epidemiology of DKA were either derived from longitudinal cohort studies that do not reveal population-based estimates or they were focused on either pediatric or adult patient populations, lacking an entire picture.

Hence, in this nationwide cohort study, we first aimed to investigate the risk of DKA in patients with type 1 and type 2 diabetes mellitus throughout lifespan and to assess sex-specific differences, time trends and relevant clinical outcomes.

## Material and methods

### Study design

This was a nationwide retrospective cohort study in pediatric, adolescent, and adult patients who were hospitalized with DKA in Switzerland between 2010 and 2018.

Hospitalization data were obtained from population-based administrative claims data provided by the Swiss Federal Office for Statistics (Bundesamt für Statistik, Neuchâtel, Switzerland). The database includes all Swiss inpatient discharge records from acute care-, general-, and specialty hospitals in Switzerland for both pediatric and adult patients. Individual-level data on patient demographics, healthcare utilization, hospital typology, medical diagnoses, diagnostic tests, clinical procedures, and in-hospital patient outcomes were provided for all hospitalized patients in Switzerland. The data were unidentifiable due to a multiple-step pseudonymization procedure. Each hospitalization in this database was identified uniquely so that re-hospitalizations could be tracked. Medical diagnoses were coded using the International Classification of Disease version 10, German Modification (ICD-10 GM) codes (http://www.who.int/classifications/icd/en/). Ethics committee Northwest and Central Switzerland (EKNZ) approved this study and granted a waiver of informed consent (Req-2021-01397).

### Case ascertainment and patient population

Eligible individuals included pediatric, adolescent, and adult persons up to an age of 90 years hospitalized with DKA and the diagnosis of either type 1 or type 2 diabetes mellitus, respectively. Cases with DKA were identified by applying the ICD-10-GM codes E10.11, E11.11. The diagnosis of diabetes mellitus was identified by the codes E10.xx for type 1 diabetes and E11.xx for type 2 diabetes, respectively.

This study followed the Strengthening The Reporting of OBservational studies in Epidemiology (STROBE) reporting guideline (19). Data on the population size per age and year were obtained from census data from the Swiss Federal Office for Statistics.

### Outcomes

The primary outcome was the incidence rate of DKA per 100,000 person-years and accompanying 95% confidence intervals (CIs) overall and stratified by age category, and sex. Secondary outcomes comprised assessment of time trends in DKA incidence and occurrence of clinical endpoints: intensive care unit (ICU) admission rate, intubation rate, length of ICU stay, incidence of cerebral edema, total length of hospital stay (LOS) – defined as days spent in the hospital during the hospitalization, all-cause in-hospital mortality, 30-day, 1-year, and 2-year all-cause hospital readmission rates. These analyses were stratified by age categories, sex, and type of diabetes mellitus.

### Statistical analysis

Unless stated otherwise, categorical variables are expressed as number (percentage) and continuous variables as mean (standard deviation, SD). DKA incidence rates are shown per 100,000 person-years and were calculated as the number of individuals with an incident DKA event divided by the sum of “person-time” population at risk in Switzerland, represented by the population size multiplied by the duration of follow-up per age and sex. The denominator for all incidence rates was the standard population per year. Exposure time began on January 1st 2010 and ended on December 31st of the cohort year 2018. Incidence rates were reported in five age categories: children (0-9 years), adolescents (10-19 years), and adults (20-29, 30-59, and 60-90 years), respectively. Differences between incidence rates were compared by calculating the 95% CI for the rate differences among sex and type of diabetes mellitus. Graphical depiction of incidence rates over age was performed using locally estimated scatterplot smoothing (LOESS).

To assess whether incidence rates of DKA changed with time, we used a mixed method linear regression model for three time periods 2010-2012, 2013-2015 and 2016-2018. For analyses on in-hospital burden, estimates of the effect sizes and corresponding 95% confidence intervals (CI) were determined using linear, logistic, or Cox proportional-hazards regressions as appropriate. All tests were 2-sided, *p <*0.05 was considered significant, and 95% confidence intervals (CIs) were reported for all incidence estimates and differences in these estimates. All statistical analyses were performed using STATA, version 15.1 (StataCorp LLC).

## Results

### Patient characteristics

From January 1^st^ 2010 to December 31^st^ 2018, a total of 5,544 hospital admissions for DKA were identified in Switzerland, yielding in an average total incidence rate of 7.46 per 100,000 person-years. Of those, 3,847 DKA events occurred in patients with type 1 diabetes and 1,697 events in patients with type 2 diabetes. The clinical characteristics of the study population are outlined in **Table 1**. Among children, adolescents, and younger adults up to an age of 29 years, nearly all cases (97.2%) with DKA were seen in patients with metabolically decompensated type 1 diabetes. While in middle-aged adults (30-59 years), 60.8% of hospitalized cases with DKA were observed in patients with type 1 diabetes, only 25.4% of cases were linked to type 1 diabetes in older adults (≥60 years). Microvascular complications of diabetes were rare among children and adolescents (0.8%), but were more prevalent in middle-aged (12.9%) and older adults (17.4%) (definition in **Supplementary Table S5**). Psychiatric disorders were seen in 11.3% of adolescents who presented with a DKA event and were diagnosed among one-third of adult patients who had an event. While in adolescents anxiety, stress-related and somatoform disorders were most prevalent, in adults the main cause of psychiatric disorder was dementia.

**Table 1:** - Baseline characteristics.

	Age 0-9 years	Age 10-19 years	Age 20-29 years	Age 30-59 years	Age 60-90 years
Number of hospitalizations, n	627	1,217	781	1,565	1,354
**Socio-demographics**					
Age, mean (SD)	5.0 (2.8)	14.7 (2.8)	23.6 (2.8)	45.7 (8.4)	73.1 (8.3)
Male gender, n (%)	342 (54.5)	552 (45.4)	401 (51.3)	942 (60.2)	677 (50.0)
Swiss citizenship, n (%)	419 (66.8)	829 (68.1)	515 (65.9)	1,036 (66.2)	1,134 (83.8)
**Type of diabetes mellitus, n (%)**					
Type 1 diabetes	616 (98.2)	1,198 (98.4)	737 (94.4)	952 (60.8)	344 (25.4)
Type 2 diabetes	11 (1.8)	19 (1.6)	44 (5.6)	613 (39.2)	1,010 (74.6)
**Comorbidities, n (%)**					
Hypertension	0 (0.0)	4 (0.3)	25 (3.2)	431 (27.5)	732 (54.1)
Dyslipidemia	3 (0.5)	11 (0.9)	22 (2.8)	261 (16.7)	269 (19.9)
Microvascular complications of diabetes mellitus	6 (1.0)	9 (0.7)	47 (6.0)	202 (12.9)	236 (17.4)
Coronary artery disease	0 (0.0)	0 (0.0)	0 (0.0)	116 (7.4)	350 (25.8)
Cerebrovascular disease	0 (0.0)	2 (0.2)	0 (0.0)	27 (1.7)	95 (7.0)
Heart failure	0 (0.0)	0 (0.0)	1 (0.1)	31 (2.0)	155 (11.4)
Peripheral artery disease	0 (0.0)	0 (0.0)	0 (0.0)	26 (1.7)	75 (5.5)
Chronic kidney disease	10 (1.6)	32 (2.6)	70 (9.0)	309 (19.7)	559 (41.3)
Psychiatric disorder	16 (2.6)	137 (11.3)	154 (19.7)	507 (32.4)	446 (32.9)

SD, standard deviation.

### Age distribution of DKA

**Figure 1:**
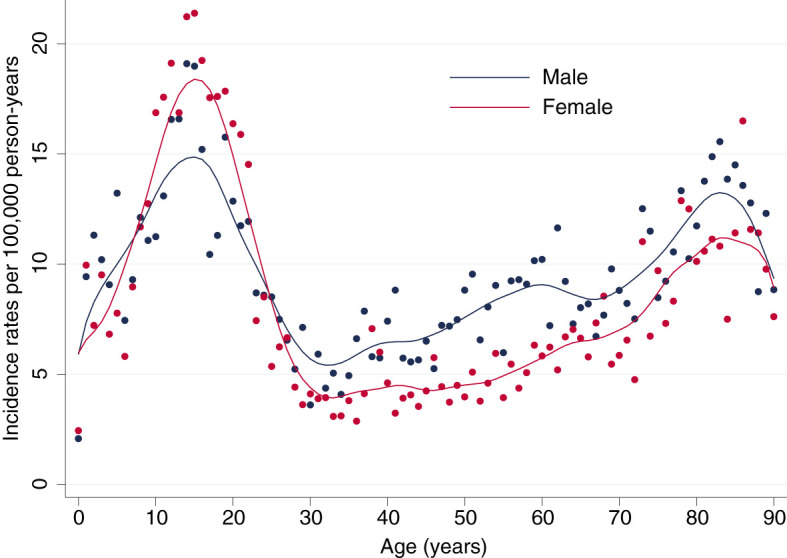
Lifetime Incidence Rates of Diabetic Ketoacidosis by Sex. Incidence rates per 100,000 person-years for male (blue) and female (red) patients.

**Figure 2:**
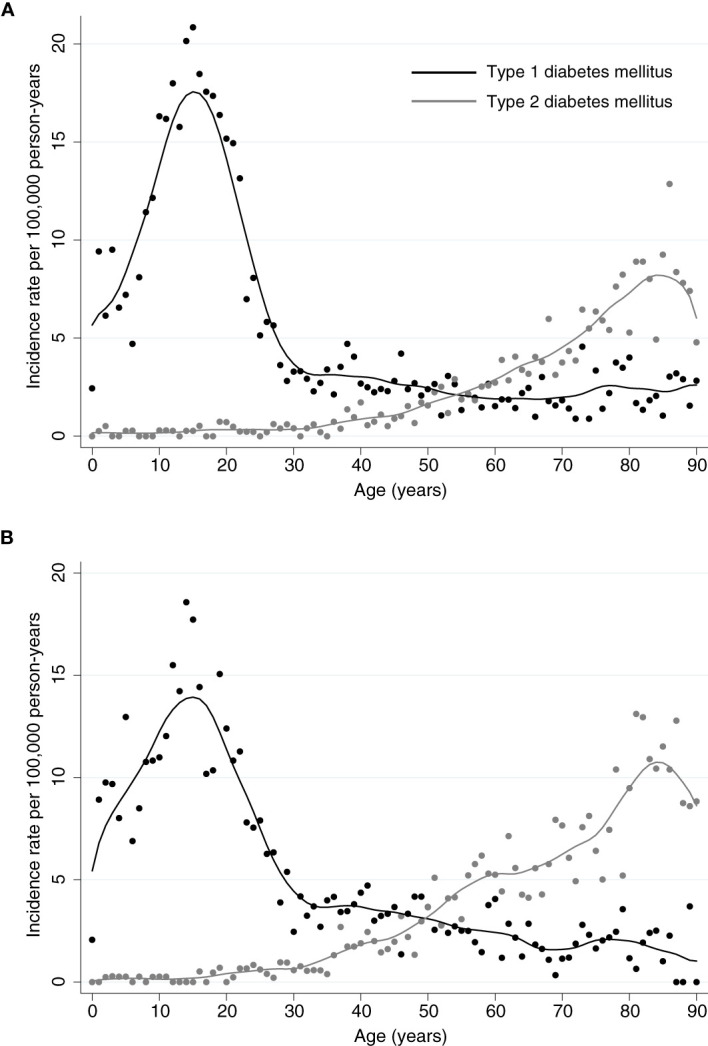
Incidence Rates of Diabetic Ketoacidosis. Incidence rates for diabetic ketoacidosis per 100,000 person-years for **(A)** female or **(B)** male patients with type 1 or type 2 diabetes, respectively. For both sexes, highest incidences for patients with type 1 diabetes occur within the adolescence and remain low from the age of 30 years. In patients with type 2 diabetes, incidences for ketoacidosis are lower and slowly increase with age from 30 with a peak around the age of 85 years.

The total incidence of DKA was highest among patients with type 1 diabetes, for both sexes incidence rates of DKA showed a steep rise from childhood to adolescence with a peak at an age of 16 years in females with a maximum of 20.84 events per 100,000 person-years and at 15 years in males with a maximum of 18.57 events per 100,000 person-years (**Figures 1**, **2**). There was a significant incidence rate difference between female and male adolescents aged 10-19 years of -3.80 events (95% CI; -5.59 to -2.02) per 100,000 person-years (**Supplementary Table S2**). Thereafter, we observed a continuous decline in rates of DKA for both sexes, reaching a low plateau at incidence rates <5 events per 100,000 person-years from the age of 30 years until advanced age.

In contrast, among patients with type 2 diabetes incidence rates of DKA were low up to an age of 37 years (<1 event per 100,000 person-years) and from then continuously rose to a maximum of 12.86 events per 100,000 person-years for females at the age of 87 years and 13.11 per 100,000 person-years for males at the age of 82 years (**Figures 1**, **2**).

### In-hospital burden

There were no clinically relevant differences in in-hospital outcomes between sexes or type of diabetes. Hospitalization with DKA - irrespective of type of diabetes - was associated with a high burden of disease and utilization of health-care, reflected by a high rate of ICU admission of 38.1% in children and of 55.5% in adolescents up to an age of 19 years. DKA was associated with cerebral edema in 0.4% of cases and with an in-hospital mortality rate of 0.1%. During the study period, among children aged below 9 years only one boy with DKA died. In this case, cerebral edema was observed and could be the underlying cause of death. These patients were also at high risk for hospital readmission within 1 year at a rate of 22.0% (**Supplementary Table S3**).

In adults aged 20 years or older, DKA was associated with a mean in-hospital mortality rate of 3.9% for both sexes and types of diabetes. Length of hospital stay was long in patients with DKA, with longest hospitalizations among the oldest age group (60-90 years) of 13.1 days (standard difference [SD] 13.4) in patients with type 1 diabetes and 11.0 days (SD 9.2) in type 2 diabetes, respectively (**Supplementary Table S3**).

### Time trends in incidence rates

**Figure 3:**
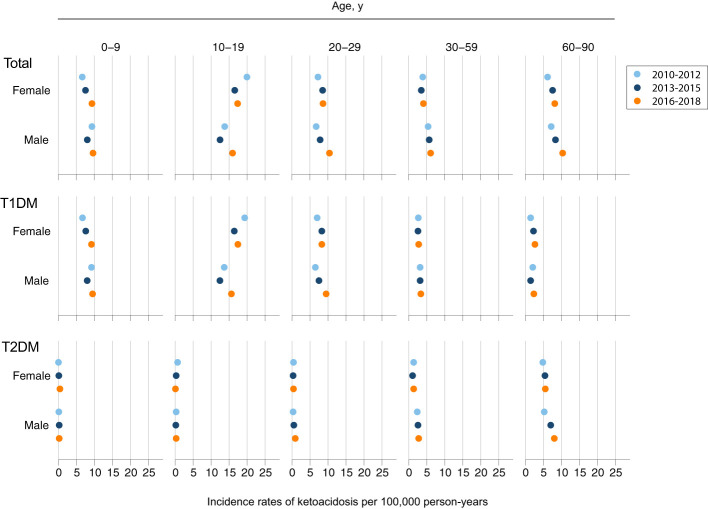
Trends of Ketoacidosis Incidence by Age, Sex, and Type of Diabetes. Shown are incidence rates per 100,000 person-years for an event of diabetic ketoacidosis for three time periods: 2010-2012 (light blue); 2013-2015 (dark blue); 2016-2018 (orange).

Incidence rates of DKA increased from 7.22 per 100,000 person-years in 2010 to 9.49 per 100,000 person-years in 2018 (**Supplementary Figure S1**). Analysis of time trends in incidence of DKA among patients with type 1 diabetes revealed that over the 9-year time frame, there was a significant increase in incidence rates among male patients aged 20-29 years (*p*<0.001) and in elderly female patients aged 60-90 years (*p*<0.001). In patients with type 2 diabetes there was an increase in DKA cases among elderly male patients aged 60-90 years (*p*=0.02), (**Figure 3** and **Supplementary Table S1**).

## Discussion

This population-based cohort study has three key findings: First, while among patients with type 1 diabetes the risk for DKA was highest among adolescents at around 15 years of age, in patients with type 2 diabetes the lifetime risk for DKA steadily increased with age. Second, in adolescence, females were predominantly prone to develop DKA, however in adults there was a switch towards higher rates in males. Third, incidence rates for DKA were increasing over time, especially among the elderly with relevant health-care burden.

So far, few data exist about the lifetime risks for DKA among patients with diabetes. While patients with diabetes are usually managed in an outpatient care setting, DKA is a life-threatening condition and requires emergency admission and most often relies on inpatient acute care. Therefore, incidence rates and in-hospital mortality rates reflect the real-world data with high certainty using nationwide hospital claims data. A previous meta-analysis investigating the incidences of DKA among patients with type 1 diabetes mellitus reported a very wide range in incidence rates of DKA ranging from 0 to 26,300 events per 100,000 person-years, however, almost all included studies were susceptible to potential selection bias or were of limited generalizability (20).

We found that in type 1 diabetes, the risks for the development of DKA were most pronounced during adolescence and in particular among girls. This finding is in line with previous data on HbA1c trajectories among youth with type 1 diabetes. Several longitudinal studies have shown that worsening of glycemic control during puberty is common and that girls were more likely to have significant deterioration of their glycemic control (21–24). Underlying factors for this gender disparity have been discussed extensively. Potential contributors to poor glucose control during puberty being more predominant in females include poorer diabetes acceptance, psychiatric disorders (e.g. depression, personality disorders), eating disorders (e.g. patients omitting inulin to induce weight loss), cognitive problems (e.g. attention deficit), binge alcohol consumption, and hazardous and risk-taking behavior inherent to adolescence (1, 25–27). In addition, increased autonomy in the management of diabetes with refusal of further parental support and the strong need to be accepted outside the family, especially by the peer group, result in less stringent diabetes treatment. Finally, somatic factors such as increased insulin requirements due to changing insulin sensitivity during puberty have an impact on glycemic control and differ between females and males (28–30). In addition, previous DKA episodes are well known risk factors for repeated DKA events in adolescents (1). Transition from pediatric to adult care during this sensitive developmental phase might further increase the risk for DKA by disengagement for clinical care of diabetes, while structured transition programs and young adult diabetes clinics show in some studies the potential to improve metabolic control and psychosocial well-being during the intervention (31–33). In summary, the DKA peak between 10-20 years reflects the multifactorial difficulties in chronic disease acceptance and management during this vulnerable developmental time window and underlines the importance of awareness for age specific multidisciplinary patient care (25).

In comparison, higher rates of DKA among males in adult age may be reflected by higher prevalence of type 2 diabetes with worse metabolic control and higher rates of obesity in men when compared with women (34). DKA no longer can be considered pathognomonic of type 1 diabetes, since a substantial number of DKA episodes especially in adult patients occur with a history of type 2 diabetes. In type 2 diabetes, DKA is known to occur commonly among patients with low social status from urban populations with high rates of obesity (35). Previous studies identified risk factors for DKA such as lack of adherence to therapy, low socioeconomic status, substance abuse, and low education (12). It has been shown that in patients with type 2 diabetes DKA is more severe with worse outcomes and higher mortality (36, 37).

It can only be speculated whether in older patients hospitalized with DKA the high rate of organic mental disorders, i.e. various forms of dementia, may be related to the fact that patients with dementia more often forget their insulin application or if patients with recurrent DKA episodes and possibly worse glycemic control are at higher risk to develop dementia. It is well established that cognitive impairment is a long-term comorbidity of diabetes mellitus (38). In addition, it has been demonstrated that in adults with type 1 diabetes, recurrent DKA events were associated with lower global cognitive function (39). Hence, approaches to prevent, early diagnose and to manage diabetes-associated cognitive impairments become increasingly important with longevity and ageing of populations.

During the 9-year study period, the population-based incidence rate of DKA increased over time. This is an intriguing finding. It may be explained by the epidemic rise in cases with type 2 diabetes or it could as well be associated with the increasing prescription rate of SGLT2 inhibitors that are widely recommended in current guidelines but are known to increase the risk of euglycemic ketoacidosis (40, 41). On the other hand, incidence rates of type 1 diabetes in children have been also increasing over time (42). There is a significant global variation in rates of DKA, being highest in developing countries (24, 43–49), which may be explained by lower disease awareness, and as a consequence delayed diagnosis (50).

Our data confirm the high in-hospital burden of DKA with a high utilization of health-care resources. While length of hospital stay might be influenced by other co-morbidities among adults, rates of ICU admissions among children may even have been underestimated since many Swiss hospitals do not provide pediatric intensive care, but rather intermediate care that was not captured in the hospital claims dataset.

### Limitations

Our data must be interpreted in the context of the study design. First and foremost, in Switzerland, having a decentralized health-care system steered by their 26 cantons (federal states), there is a strong need for improved completeness, accessibility, and linkability of health-care data. For instance, there is minimal information on the incidence of type 1 and of type 2 diabetes within the Swiss population, hence incidence rates in our study were calculated in the general population and not in patients with diabetes only. Second, our study is based on administrative hospital claims data that do neither contain information on the duration of diabetes - thus DKA events may have occurred in newly diagnosed as well as in established disease – nor do they contain medication data (e.g. insulin pump usage). Unfortunately, in Switzerland there is no national database on utilization of medications. Third, data on causes of ICU admission, mechanical ventilation, and death were not available. Fourth, data on laboratory parameters such as glycemic control were likewise not available, therefore associations of DKA with poor glycemic control cannot be assessed in our study. Finally, we cannot exclude a certain risk of misclassification and underreporting since administrative data were used in our analyses and we were not able to validate diagnoses, as a consequence it is possible that patients with latent autoimmune diabetes in adults (LADA) may have been misclassified. Nevertheless, a previous validation study verified that the diagnosis of DKA based on hospital claims data had a positive predictive value of around 90% (88.9%, 95% CI; 71.9 to 96.1%) (51).

There are several strengths of note: Our analysis was based on nationwide hospital care data with high external validity, a strong power, and it encompasses a long study period. Furthermore, due to the fact that DKA almost always requires hospitalization, our data allow for highly accurate estimates at the national level. The results highlight the need for further studies on national incidences of DKA especially from countries, where national estimates of diabetes prevalence exist and where linkage to other registries, such as medication usage are available, in order to decipher causes and evolve possible strategies to combat the trend of rising DKA incidences.

In conclusion, DKA is a life-threatening complication of diabetes that can occur at all ages. This study characterizes subpopulations at highest risk for DKA, which showed an overall increase over time with a high burden of disease and significant health-care utilization. Our results highlight the need for intensified efforts to optimize ambulatory care with incorporation of educational, clinical, and social support and to individualize transition from pediatric to adult care clinics. Most likely such an approach has to be both interdisciplinary and interprofessional.

## Data availability statement

The data analyzed in this study is subject to the following licenses/restrictions: The data that support the findings of this study are available from the Swiss Federal Office for Statistics. Restrictions apply to the availability of these data, which were used under license for this study. Requests to access these datasets should be directed to Swiss Federal Office for Statistics.

## Author contributions

FE, AK, and GS designed the study and wrote the manuscript. FE and AK analyzed the data and were responsible for the decision to submit the manuscript. All authors provided substantial comments on drafts and approved the final report.

## Funding

This study was supported by the Kantonsspital Aarau AG. The funding sources had no role in study design, data collection, data analysis, data interpretation, or writing of the report.

## Acknowledgments

We thank the Swiss Federal Office for Statistics (Bundesamt für Statistik, Neuchâtel, Switzerland) for the acquisition and provision of data.

## Conflict of interest

The authors declare that the research was conducted in the absence of any commercial or financial relationships that could be construed as a potential conflict of interest.

## Publisher’s note

All claims expressed in this article are solely those of the authors and do not necessarily represent those of their affiliated organizations, or those of the publisher, the editors and the reviewers. Any product that may be evaluated in this article, or claim that may be made by its manufacturer, is not guaranteed or endorsed by the publisher.
