# Supplementary material for: Lifetime risk and health-care burden of diabetic ketoacidosis: A population-based study
Source: Front Endocrinol (Lausanne). 2022 Aug 24;13:940990. doi: 10.3389/fendo.2022.940990 (PMC9449722; doi:10.3389/fendo.2022.940990)
Supplement: Supplementary file 1 [file DataSheet_1.docx]

Supplementary Appendix

This appendix has been provided by the authors to give readers additional information about their work.

Supplement to: Ebrahimi F and Kutz A et al. Lifetime Risk and Health-Care Burden of Diabetic Ketoacidosis: *a population-based study*

**Table S1: Time trends in incidence rates of diabetic ketoacidosis**

| **Age category** | | | **Age 0-9 years** | | | **Age 10-19 years** | | | **Age 20-29 years** | | | **Age 30-59 years** | | | **Age 60-90 years** | | |
| --- | --- | --- | --- | --- | --- | --- | --- | --- | --- | --- | --- | --- | --- | --- | --- | --- | --- |
| **Type of DM** | **Sex** | **Time period** | **Event, n** | **Incidence rate** | **p value** | **Event, n** | **Incidence rate** | **p value** | **Event, n** | **Incidence rate** | **p value** | **Event, n** | **Incidence rate** | **p value** | **Event, n** | **Incidence rate** | **p value** |
| **Total** | Female | 2010-2012 | 76 | 6.64 | 0.10 | 250 | 19.92 | 0.67 | 111 | 7.36 | 0.19 | 206 | 3.99 | 0.63 | 183 | 6.27 | <0.001 |
|  |  | 2013-2015 | 91 | 7.58 |  | 203 | 16.52 |  | 133 | 8.57 |  | 192 | 3.60 |  | 234 | 7.67 |  |
|  |  | 2016-2018 | 118 | 9.41 |  | 212 | 17.34 |  | 136 | 8.74 |  | 225 | 4.11 |  | 260 | 8.14 |  |
|  | Male | 2010-2012 | 112 | 9.27 | 0.43 | 183 | 13.85 | 0.06 | 105 | 6.78 | <0.001 | 286 | 5.46 | 0.03 | 175 | 7.25 | 0.003 |
|  |  | 2013-2015 | 102 | 8.04 |  | 162 | 12.51 |  | 127 | 7.96 |  | 312 | 5.73 |  | 217 | 8.43 |  |
|  |  | 2016-2018 | 128 | 9.66 |  | 207 | 15.93 |  | 169 | 10.41 |  | 344 | 6.18 |  | 285 | 10.36 |  |
| **T1DM** | Female | 2010-2012 | 76 | 6.64 | 0.15 | 242 | 19.28 | 0.79 | 105 | 6.96 | 0.18 | 138 | 2.67 | 0.42 | 43 | 1.47 | <0.001 |
|  |  | 2013-2015 | 90 | 7.50 |  | 201 | 16.36 |  | 128 | 8.24 |  | 134 | 2.51 |  | 68 | 2.23 |  |
|  |  | 2016-2018 | 114 | 9.09 |  | 212 | 17.34 |  | 130 | 8.35 |  | 154 | 2.82 |  | 84 | 2.63 |  |
|  | Male | 2010-2012 | 111 | 9.19 | 0.49 | 180 | 13.62 | 0.06 | 100 | 6.46 | <0.001 | 162 | 3.09 | 0.09 | 49 | 2.03 | 0.87 |
|  |  | 2013-2015 | 100 | 7.88 |  | 160 | 12.35 |  | 119 | 7.46 |  | 174 | 3.20 |  | 36 | 1.40 |  |
|  |  | 2016-2018 | 125 | 9.43 |  | 203 | 15.62 |  | 155 | 9.55 |  | 190 | 3.42 |  | 64 | 2.33 |  |
| **T2DM** | Female | 2010-2012 | 0 | 0.00 | 0.001 | 8 | 0.64 | 0.07 | 6 | 0.40 | 0.79 | 68 | 1.32 | 0.84 | 140 | 4.80 | 0.11 |
|  |  | 2013-2015 | 1 | 0.08 |  | 2 | 0.16 |  | 5 | 0.32 |  | 58 | 1.09 |  | 166 | 5.44 |  |
|  |  | 2016-2018 | 4 | 0.32 |  | 0 | 0.00 |  | 6 | 0.39 |  | 71 | 1.30 |  | 176 | 5.50 |  |
|  | Male | 2010-2012 | 1 | 0.08 | 0.38 | 3 | 0.23 | 0.85 | 5 | 0.32 | 0.03 | 124 | 2.37 | 0.16 | 126 | 5.22 | 0.02 |
|  |  | 2013-2015 | 2 | 0.16 |  | 2 | 0.15 |  | 8 | 0.50 |  | 138 | 2.54 |  | 181 | 7.03 |  |
|  |  | 2016-2018 | 3 | 0.23 |  | 4 | 0.31 |  | 14 | 0.86 |  | 154 | 2.77 |  | 221 | 8.03 |  |

**Table S2 –** **Incidence rates of diabetic ketoacidosis among age category and sex, stratified by type of diabetes mellitus**

|  | | **Age 0-9 years** | | **Age 10-19 years** | | **Age 20-29 years** | | **Age 30-59 years** | | **Age 60-90 years** | | |
| --- | --- | --- | --- | --- | --- | --- | --- | --- | --- | --- | --- | --- |
|  | | **Female** | **Male** | **Female** | **Male** | **Female** | **Male** | **Female** | **Male** | **Female** | **Male** |  |
| **T1DM** | Number of events | 280 | 336 | 655 | 543 | 363 | 374 | 426 | 526 | 195 | 149 |  |
|  | Follow-up, PY | 3’597’873 | 3’801’954 | 3’706’677 | 3’915’965 | 4’616’431 | 4’766’589 | 15’979’027 | 16’252’240 | 9’164’900 | 7’739’576 |  |
|  | Incidence rate, per 100,000 PY | 7.78 | 8.84 | 17.67 | 13.87 | 7.86 | 7.85 | 2.67 | 3.24 | 2.13 | 1.93 |  |
|  | Incidence rate difference (95% CI) | − | 1.06 (-0.26 to 2.37) | − | -3.80 (-5.59 to -2.02) | − | -0.02 (-1.15 to 1.12) | − | 0.57 (0.20 to 0.95) | − | -0.20 (-0.63 to 0.23) |  |
| **T2DM** | Number of events | 5 | 6 | 10 | 9 | 17 | 27 | 197 | 416 | 482 | 528 |  |
|  | Follow-up, PY | 3’597’873 | 3’801’954 | 3’706’677 | 3’915’965 | 4’616’431 | 4’766’589 | 15’979’027 | 16’252’240 | 9’164’900 | 7’739’576 |  |
|  | Incidence rate, per 100,000 PY | 0.14 | 0.16 | 0.27 | 0.23 | 0.37 | 0.57 | 1.23 | 2.56 | 5.26 | 6.82 |  |
|  | Incidence rate difference (95% CI) | − | 0.02 (-0.16 to 0.19) | −] | -0.04 (-0.27 to 0.19) | − | 0.20 (-0.08 to 0.47) | − | 1.33 (1.03 to 1.63) | − | 1.56 (0.82 to 2.31) |  |
| **Total** | Number of events | 285 | 342 | 665 | 552 | 380 | 401 | 623 | 942 | 677 | 677 |  |
|  | Follow-up, PY | 3’597’873 | 3’801’954 | 3’706’677 | 3’915’965 | 4’616’431 | 4’766’589 | 15’979’027 | 16’252’240 | 9’164’900 | 7’739’576 |  |
|  | Incidence rate, per 100,000 PY | 7.92 | 9.00 | 17.94 | 14.10 | 8.23 | 8.41 | 3.90 | 5.80 | 7.39 | 8.75 |  |
|  | Incidence rate difference (95% CI) | − | 1.07 (-0.25 to 2.40) | − | -3.84 (-5.65 to -2.04) | − | 0.18 (-0.99 to 1.35) | − | 1.90 (1.42 to 2.38) | −] | 1.36 (0.50 to 2.22) |  |

For calculation of incidence rate differences between males and females, incidence rates in females were defined as reference. Abbreviations: CI, confidence interval; PY, person-years; T1DM, type 1 diabetes mellitus; T2DM, type 2 diabetes mellitus.

**Table S3 - Patient outcomes stratified by age, gender, and type of diabetes mellitus**

|  | | **Age 0-9 years** | | **Age 10-19 years** | | **Age 20-29 years** | | **Age 30-59 years** | | **Age 60-90 years** | | |
| --- | --- | --- | --- | --- | --- | --- | --- | --- | --- | --- | --- | --- |
| **T1DM** | | **Female**  **(n=280)** | **Male**  **(n=336)** | **Female**  **(n=655)** | **Male**  **(n=543)** | **Female**  **(n=363)** | **Male**  **(n=374)** | **Female**  **(n=426)** | **Male**  **(n=526)** | **Female**  **(n=195)** | **Male**  **(n=149)** |  |
| **ICU admission** | n (%) | 89 (31.8) | 93 (27.7) | 283 (43.2) | 222 (40.9) | 202 (55.7) | 216 (57.8) | 254 (59.6) | 321 (61.0) | 120 (61.5) | 85 (57.1) |  |
|  | OR (95% CI) | 1 [Reference] | 0.82 (0.58 to 1.16) | 1 [Reference] | 0.91 (0.72 to 1.14) | 1 [Reference] | 1.09 (0.81 to 1.46) | 1 [Reference] | 1.06 (0.82 to 1.38) | 1 [Reference] | 0.83 (0.54 to 1.28) |  |
| **Mechanical ventilation** | n (%) | 2 (0.7) | 4 (1.2) | 4 (0.6) | 5 (0.9) | 6 (1.7) | 4 (1.1) | 26 (6.1) | 31 (5.9) | 17 (8.7) | 15 (10.1) |  |
|  | OR (95% CI) | 1 [Reference] | 1.67 (0.30 to 9.21) | 1 [Reference] | 1.51 (0.40 to 5.66) | 1 [Reference] | 0.64 (0.18 to 2.30) | 1 [Reference] | 0.96 (0.56 to 1.65) | 1 [Reference] | 1.17 (0.57 to 2.43) |  |
| **Length of ICU stay (d)** | mean (SD) | 1.2 (0.6) | 1.4 (0.8) | 1.7 (1.1) | 1.6 (1.6) | 2.1 (2.3) | 1.8 (1.1) | 2.6 (2.3) | 2.3 (2.6) | 2.7 (3.3) | 4.6 (5.9) |  |
|  | Cf. (95% CI) | − | 0.22 (-0.01 to 0.45) | − | -0.06 (-0.30 to 0.18) | − | -0.34 (-0.69 to 0.01) | − | -0.23 (-0.66 to 0.20) | −] | 1.85 (0.55 to 3.14) |  |
| **Cerebral edema** | n (%) | 1 (0.4) | 0 (0.0) | 3 (0.5) | 4 (0.7) | 1 (0.3) | 0 (0.0) | 1 (0.2) | 0 (0.0) | 0 (0.0) | 0 (0.0) |  |
|  | OR (95% CI) | 1 [Reference] | N/A | 1 [Reference] | 1.61 (0.36 to 7.24) | 1 [Reference] | N/A | 1 [Reference] | N/A | 1 [Reference] | N/A |  |
| **Length of hospital stay (d)** | mean (SD) | 9.5 (5.6) | 8.1 (6.5) | 6.7 (12.5) | 6.8 (20.0) | 5.2 (5.0) | 4.9 (3.7) | 7.4 (7.3) | 7.2 (7.3) | 12.0 (11.3) | 13.1 (13.4) |  |
|  | Cf. (95% CI) | − | -1.39 (-2.36 to -0.42) | − | 0.04 (-1.83 to 1.90) | − | -0.32 (-0.95 to 0.31) | − | -0.27 (-1.20 to 0.66) | − | 1.02 (-1.59 to 3.64) |  |
| **In-hospital mortality** | n (%) | 0 (0.0) | 1 (0.3) | 0 (0.0) | 0 (0.0) | 1 (0.3) | 0 (0.0) | 1 (0.2) | 3 (0.6) | 14 (7.2) | 10 (6.7) |  |
|  | OR (95% CI) | 1 [Reference] | N/A | 1 [Reference] | N/A | 1 [Reference] | N/A | 1 [Reference] | 2.44 (0.25 to 23.52) | 1 [Reference] | 0.93 (0.40 to 2.16) |  |
| **30-day readmission** | n (%) | 7 (2.5) | 10 (3.0) | 17 (2.6) | 13 (2.4) | 13 (3.6) | 10 (2.7) | 22 (5.2) | 27 (5.1) | 14 (7.2) | 8 (5.4) |  |
|  | OR (95% CI) | 1 [Reference] | 1.20 (0.45 to 3.18) | 1 [Reference] | 0.92 (0.44 to 1.91) | 1 [Reference] | 0.74 (0.32 to 1.71) | 1 [Reference] | 0.99 (0.56 to 1.77) | 1 [Reference] | 0.73 (0.30 to 1.80) |  |
| **1-year readmission** | n (%) | 43 (15.4) | 67 (19.9) | 174 (26.6) | 120 (22.1) | 98 (27.0) | 84 (22.5) | 109 (25.6) | 143 (27.2) | 60 (30.8) | 45 (30.2) |  |
|  | OR (95% CI) | 1 [Reference] | 1.37 (0.90 to 2.09) | 1 [Reference] | 0.78 (0.60 to 1.02) | 1 [Reference] | 0.78 (0.56 to 1.10) | 1 [Reference] | 1.09 (0.81 to 1.45) | 1 [Reference] | 0.97 (0.61 to 1.55) |  |
| **2-year readmission** | n (%) | 62 (22.1) | 98 (29.2) | 225 (34.4) | 151 (27.8) | 137 (37.7) | 116 (31.0) | 143 (33.6) | 176 (33.5) | 74 (38.0) | 57 (38.3) |  |
|  | OR (95% CI) | 1 [Reference] | 1.45 (1.00 to 2.09) | 1 [Reference] | 0.74 (0.57 to 0.94) | 1 [Reference] | 0.74 (0.55 to 1.01) | 1 [Reference] | 1.0 (0.76 to 1.30) | 1 [Reference] | 1.01 (0.65 to 1.57) |  |
|  | | | | | | | | | | | | |
| **T2DM** | | **Female**  **(n=5)** | **Male**  **(n=6)** | **Female**  **(n=10)** | **Male**  **(n=9)** | **Female**  **(n=17)** | **Male**  **(n=27)** | **Female**  **(n=197)** | **Male**  **(n=416)** | **Female**  **(n=482)** | **Male**  **(n=528)** |  |
| **ICU admission** | n (%) | 3 (60.0) | 2 (33.3) | 7 (70.0) | 3 (33.3) | 4 (23.5) | 17 (63.0) | 108 (54.8) | 221 (53.1) | 244 (50.6) | 262 (49.6) |  |
|  | OR (95% CI) | 1 [Reference] | 0.33 (0.03 to 3.93) | 1 [Reference] | 0.21 (0.03 to 1.49) | 1 [Reference] | 5.53 (1.41 to 21.66) | 1 [Reference] | 0.93 (0.66 to 1.31) | 1 [Reference] | 0.96 (0.75 to 1.23) |  |
| **Mechanical ventilation** | n (%) | 0 (0.0) | 0 (0.0) | 1 (10.0) | 0 (0.0) | 0 (0.0) | 1 (3.7) | 17 (8.6) | 43 (10.3) | 59 (12.2) | 63 (11.9) |  |
|  | OR (95% CI) | 1 [Reference] | N/A | 1 [Reference] | N/A | 1 [Reference] | N/A | 1 [Reference] | 1.22 (0.68 to 2.20) | 1 [Reference] | 0.97 (0.67 to 1.42) |  |
| **Length of ICU stay (d)** | mean (SD) | 1.5 (0.2) | 1.2 (0.3) | 1.4 (1.2) | 3.1 (1.7) | 1.4 (0.9) | 2.1 (1.7) | 3.7 (4.6) | 3.1 (3.8) | 3.1 (4.8) | 3.3 (3.9) |  |
|  | Cf. (95% CI) | − | -0.31 (-1.39 to 0.77) | − | 1.70 (-0.39 to 3.78) | − | 0.68 (-1.18 to 2.55) | − | -0.60 (-1.57 to 0.37) | − | 0.18 (-0.59 to 0.94) |  |
| **Cerebral edema** | n (%) | 0 (0.0) | 0 (0.0) | 1 (10) | 0 (0.0) | 0 (0.0) | 0 (0.0) | 1 (0.5) | 0 (0.0) | 0 (0.0) | 1 (0.2) |  |
|  | OR (95% CI) | 1 [Reference] | N/A | 1 [Reference] | N/A | 1 [Reference] | N/A | 1 [Reference] | N/A | 1 [Reference] | N/A |  |
| **Length of hospital stay (d)** | mean (SD) | 8.8 (5.2) | 9.0 (5.5) | 3.9 (3.3) | 5.9 (4.9) | 7.6 (9.1) | 7.2 (3.7) | 10.7 (9.6) | 9.2 (8.7) | 12.2 (10.6) | 11.0 (9.2) |  |
|  | Cf. (95% CI) | − | 0.20 (-7.20 to 7.60) | − | 1.99 (-2.00 to 5.98) | − | -0.40 (-4.36 to 3.55) | − | -1.52 (-3.05 to >0.00) | − | -1.21 (-2.43 to 0.02) |  |
| **In-hospital mortality** | n (%) | 0 (0.0) | 0 (0.0) | 1 (10.0) | 0 (0.0) | 0 (0.0) | 0 (0.0) | 1 (0.5) | 11 (2.6) | 47 (9.8) | 57 (10.8) |  |
|  | OR (95% CI) | 1 [Reference] | N/A | 1 [Reference] | N/A | 1 [Reference] | N/A | 1 [Reference] | 5.32 (0.68 to 41.53) | 1 [Reference] | 1.12 (0.75 to 1.68) |  |
| **30-day readmission** | n (%) | 0 (0.0) | 0 (0.0) | 0 (0.0) | 0 (0.0) | 2 (11.8) | 2 (7.4) | 9 (4.6) | 17 (4.1) | 15 (3.1) | 38 (7.2) |  |
|  | OR (95% CI) | 1 [Reference] | N/A | 1 [Reference] | N/A | 1 [Reference] | 0.60 (0.08 to 4.72) | 1 [Reference] | 0.89 (0.39 to 2.03) | 1 [Reference] | 2.41 (1.31 to 4.45) |  |
| **1-year readmission** | n (%) | 0 (0.0) | 0 (0.0) | 1 (10.0) | 1 (11.1) | 8 (47.1) | 7 (25.9) | 42 (21.3) | 96 (23.1) | 109 (22.6) | 136 (25.8) |  |
|  | OR (95% CI) | 1 [Reference] | N/A | 1 [Reference] | 1.13 (0.06 to 21.09) | 1 [Reference] | 0.39 (0.11 to 1.42) | 1 [Reference] | 1.11 (0.73 to 1.67) | 1 [Reference] | 1.19 (0.89 to 1.59) |  |
| **2-year readmission** | n (%) | 1 (20.0) | 2 (33.3) | 3 (30.0) | 3 (33.3) | 9 (52.9) | 9 (33.3) | 55 (27.9) | 111 (26.7) | 136 (28.2) | 168 (31.8) |  |
|  | OR (95% CI) | 1 [Reference] | 2.00 (0.13 to 31.98) | 1 [Reference] | 1.17 (0.17 to 8.09) | 1 [Reference] | 0.44 (0.13 to 1.54) | 1 [Reference] | 0.94 (0.64 to 1.37) | 1 [Reference] | 1.19 (0.91 to 1.56) |  |

Mechanical ventilation rates were assessed among ICU patients only. For calculation of risk ratios/differences between males and females, females were defined as reference. Abbreviations: Cf., regression coefficient; CI, confidence interval; N/A, not applicable; OR, odds ratio; PY, person-years; T1DM, type 1 diabetes mellitus; T2DM, type 2 diabetes mellitus.

**Table S4: Characterization of psychiatric disorders among patients hospitalized with diabetic ketoacidosis**

|  | **ICD 10 code** | **Age 0-9 years** | **Age 10-19 years** | **Age 20-29 years** | **Age 30-59 years** | **Age 60-90 years** |
| --- | --- | --- | --- | --- | --- | --- |
| **Organic, including symptomatic, mental disorders , n(%)** | F00-F09 | 1 (6.2%) | 2 (1.5%) | 3 (2.0%) | 19 (3.7%) | 184 (41.3%) |
| **Mental and behavioural disorders due to psychoactive substance use , n(%)** | F10-F19 | 0 | 15 (10.9%) | 51 (33.1%) | 177 (34.9%) | 96 (21.5%) |
| **Schizophrenia, schizotypal, delusional, and other non-mood psychotic disorders, n(%)** | F20-F29 | 0 | 0 | 10 (6.5%) | 51 (10.1%) | 14 (3.2%) |
| **Mood [affective] disorders, n(%)** | F30-F39 | 0 | 14 (10.2%) | 18 (11.7%) | 132 (26.0%) | 98 (22.0%) |
| **Anxiety, dissociative, stress-related, somatoform and other nonpsychotic mental disorders, n(%)** | F40-F49 | 2 (12.5%) | 33 (24.1%) | 33 (21.4%) | 70 (13.8%) | 35 (7.8%) |
| **Behavioral syndromes associated with physiological disturbances and physical factors, n(%)** | F50-F59 | 0 | 18 (13.1%) | 9 (5.8%) | 4 (0.8%) | 1 (0.2%) |
| **Disorders of adult personality and behavior, n(%)** | F60-F69 | 0 | 14 (10.2%) | 22 (14.3%) | 33 (6.5%) | 9 (2.0%) |
| **Mental retardation , n(%)** | F70-F79 | 1 (6.2%) | 1 (0.8%) | 2 (1.3%) | 13 (2.6%) | 9 (2.0%) |
| **Disorders of psychological development , n(%)** | F80-F89 | 7 (43.8%) | 13 (9.5%) | 4 (2.6%) | 3 (0.6%) | 0 |
| **Behavioral and emotional disorders with onset usually occurring in childhood and adolescence, n(%)** | F90-F98 | 5 (31.3%) | 27 (19.7%) | 2 (1.3)% | 5 (1.0%) | 0 |
| **Total** | F00-F98 | 16 | 137 | 154 | 507 | 446 |

**Table S5: ICD code definition of microvascular disease**

|  | **ICD 10 code** |
| --- | --- |
| **Diabetic nephropathy** | E10.2x, E11.2x |
| **Diabetic retinopathy** | E10.31x, E10.32x, E10.33x, E10.34x, E10.35x, E10.37x, E11.31x, E11.32x, E11.33x, E11.34x, E11.35x, E11.37x |
| **Diabetic neuropathy** | E10.4x, E10.610, E11.4x, E11.610. |

**Figure S1 – Incidences of diabetic ketoacidosis in Switzerland
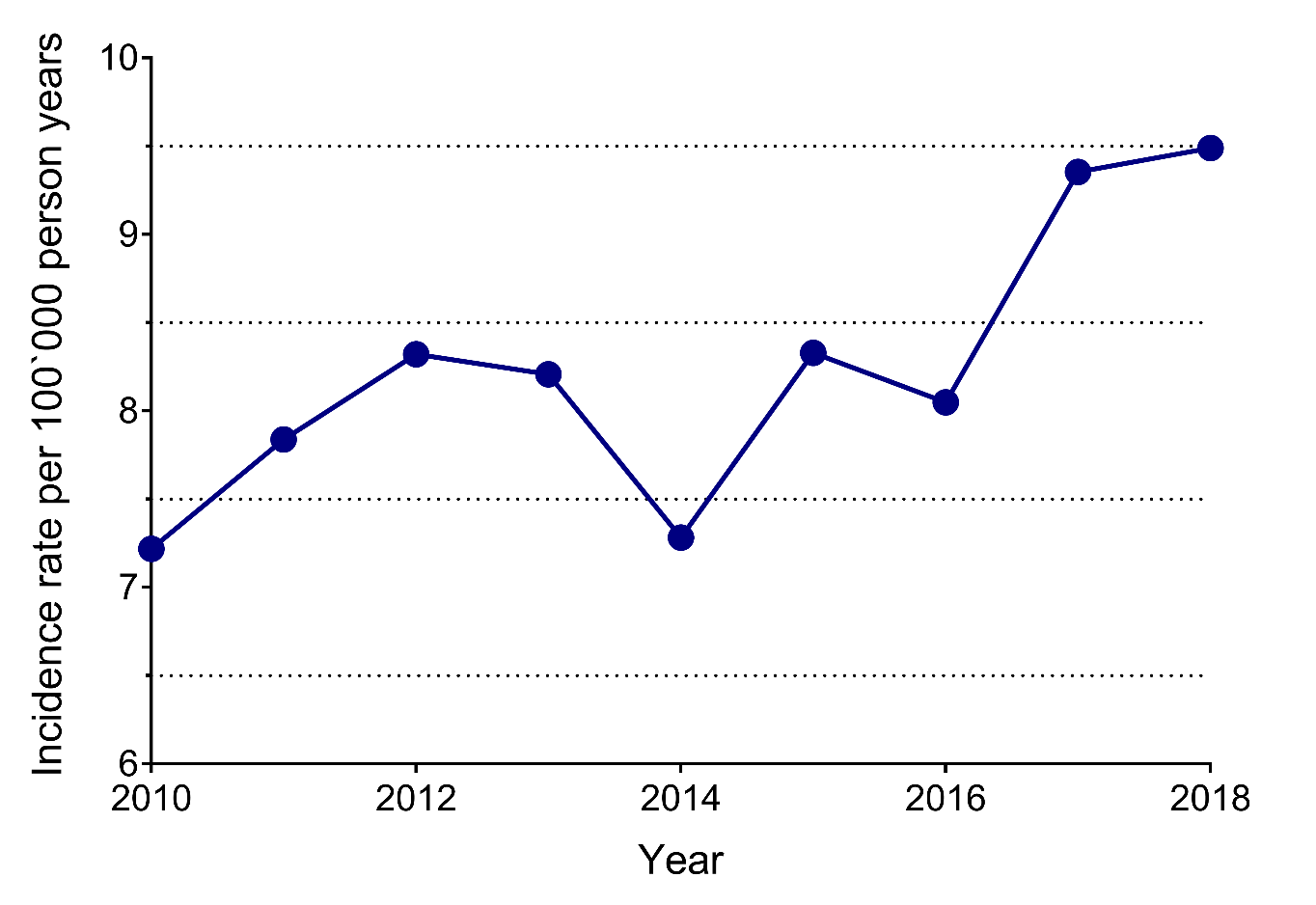
**
